# An Evidence-Based Educational Intervention for Reducing Coercive Measures in Psychiatric Hospitals: A Randomized Clinical Trial

**DOI:** 10.1001/jamanetworkopen.2022.29076

**Published:** 2022-08-30

**Authors:** Maritta Välimäki, Tella Lantta, Minna Anttila, Tero Vahlberg, Sharon-Lise Normand, Min Yang

**Affiliations:** 1 Department of Nursing Science, University of Turku, Turku, Finland; 2 Xiangya Nursing School, Xiangya Research Center of Evidence-based Healthcare, Central South University, Hunan, China; 3 Now with Faculty of Health and Education, Department of Nursing, Manchester Metropolitan University, Manchester, UK; 4 Department of Biostatistics, University of Turku, Turku, Finland; 5 Department of Health Care Policy, Harvard Medical School, Boston, Massachusetts; 6 Department of Biostatistics, Harvard T.H. Chan School of Public Health, Boston, Massachusetts; 7 West China School of Public Health, Sichuan University, Chengdu, China; 8 Faculty of Design, Health, and Art, Swinburne University of Technology, Hawthorn, Victoria, Australia

## Abstract

**Importance:**

Investing in health care staffs’ education can change the scope of action and improve care. The effectiveness of staff education remains inconclusive.

**Objective:**

To examine whether an evidence-based educational intervention for nurses decreases the use of seclusion rooms in psychiatric hospitals compared with usual practice.

**Design, Setting, and Participants:**

In this pragmatic, 2-arm parallel, stratified cluster randomized clinical trial, 28 wards in 15 psychiatric hospitals in Finland were screened for eligibility and randomly allocated (1:1). Nurses joined on either intervention (n = 13) or usual practice (n = 15) wards. The intervention was performed from May 1, 2016, to October 31, 2017. The follow-up data for January 1 to December 31, 2017, were collected from hospital registers in 2018. Data analysis was performed October 27, 2021.

**Interventions:**

Evidence-based education delivered during 18 months, including 8 months of active education, followed by a 10**-**month maintenance period.

**Main Outcomes and Measures:**

The primary outcome was the occurrence of patient seclusion (events per total number of patients).

**Results:**

Of 28 psychiatric hospital wards screened (437 beds and 648 nurses), 27 wards completed the study. A total of 8349 patients were receiving care in the study wards, with 53% male patients and a mean (SD) age of 40.6 (5.7) years. The overall number of seclusions was 1209 (14.5%) in 2015 and 1349 (16.5%) in 2017. In the intervention group, the occurrence rate of seclusion at the ward level decreased by 5.3% from 629 seclusions among 4163 patients (15.1%) to 585 seclusions among 4089 patients (14.3%) compared with a 34.7% increase from 580 seclusions among 4186 patients (13.9%) to 764 seclusions among 4092 patients (18.7%) in the usual practice group. The adjusted rate ratio was 0.86 (95% CI, 0.40-1.82) in 2015 and 0.66 (95% CI, 0.31-1.41) in 2017 (*P* = .003). However, the number of forced injections increased in the intervention group from 317 events among 4163 patients (7.6%) in 2015 to 486 events among 4089 patients (11.9%) in 2017 compared with an increase in the usual practice group from 414 events among 4186 patients (9.9%) in 2015 to 481 events among 4092 patients (11.8%) in 2017. Seven adverse events were reported.

**Conclusions and Relevance:**

In this randomized clinical trial, the educational intervention had a limited effect on the change of occurrence rate of patient seclusion, whereas the use of forced injections increased. More studies are needed to better understand the reasons for these findings.

**Trial Registration:**

ClinicalTrials.gov Identifier: NCT02724748

## Key Points

**Question:**

Does an evidence-based educational intervention for nurses decrease the use of seclusion rooms in psychiatric hospitals compared with usual practice?

**Findings:**

In this cluster randomized clinical trial of 8349 patients receiving care in 27 wards at 15 psychiatric hospitals across Finland, the use of a seclusion room at the ward level decreased from 15.1% to 14.3% in the intervention group and increased from 13.9% to 18.7% in the usual practice group.

**Meaning:**

Although feasible, the educational intervention had a limited effect for change of occurrence rate of patient seclusion.

## Teaser

This randomized clinical trial of psychiatric hospital wards in Finland examines whether an educational intervention for nurses decreases the use of seclusion rooms.

## Introduction

The use of coercive measures in psychiatric hospitals is a global concern.^1^ Arguments for using coercive measures focus on preventing patients from harming themselves and others.^2^ However, evidence supporting the benefits of coercive measures remains inconclusive^3^ because such methods can lead to physical damage, psychological distress in patients and staff,^4^ and even patient death.^5^ Patients and frontline staff have raised concerns about the quality of psychiatric inpatient care.^6^ The cycle of restrictive environments and poor inpatient care might be broken with staff training, the most common strategy used to reduce restrictive practices in mental health care.^7^

The EUNOMIA (European Evaluation of Coercion in Psychiatry and Harmonisation of Best Clinical Practice) study^8^ improved the communication between community and hospital teams and developed training courses for professionals on the management of aggressive behaviors. In line with these recommendations and the paucity of large-scale trials, we designed an educational intervention, VIOLIN (Violence Intervention), to improve treatment culture and reduce the need for the use of coercive methods in psychiatric care. We tested whether an evidence-based educational intervention decreases the use of seclusion rooms, the most used coercive measure in psychiatric hospitals,^9^ compared with usual practice. We also examined how the educational intervention affected the use of other types of coercion measures (limb restraint and forced medication); service use; nurses’ team climate; turnover; and patients’ functional capacity, treatment satisfaction, and quality of life.

## Methods

### Study Design and Participants

We conducted a 2-arm parallel, stratified cluster randomized clinical trial in government-funded psychiatric hospitals (15 hospitals with 28 wards, 1-5 wards in each hospital) located across Finland. Hospitals had least 1 adult psychiatric ward that used coercive measures as defined in the Finnish Mental Health Act^10^ and were not currently participating in a similar type of study. Hospital wards that specialized solely in forensic psychiatry, psychogeriatric care, or child and adolescent psychiatric care were excluded. All nurses (registered nurses and licensed practical nurses) working on the study wards, without any inclusion or exclusion criteria, were invited to join this intention-to-treat trial; none refused. For patients, inclusion criteria were admission to the study ward at the time of the data collection, fluency in Finnish, age of 18 years or older, and the ability and willingness to participate in an anonymous patient survey. No formal capacity test was implemented; rather, we relied on the judgment of the health care professionals. Ethical review was conducted by the Ethics Committee of Hospital District of Southwest Finland. Hospitals granted permission to conduct the study. According to Finnish regulations, a completed and returned survey form was interpreted as voluntary informed consent, whereas the data collected in interviews were based on written informed consent. We followed the Consolidated Standards of Reporting Trials (CONSORT) reporting guideline. The study protocol is published elsewhere.^11^ The trial protocol can be found in Supplement 1. Differences between the original protocol are described in eAppendix 1 in Supplement 2.

### Randomization and Masking

Hospitals were assigned in a 1:1 ratio to the intervention group (evidence-based educational intervention) or the usual practice group. To avoid contamination between staff on study wards, the randomization units were the hospitals, which were stratified by the number of nursing staff vacancies and beds on each study ward. Randomization was centralized, concealed, and computer generated by an independent statistician. Because of the type of intervention, researchers and study participants were not blinded to allocation.

### Procedures

VIOLIN is a multicomponent, 18-month educational intervention that aims to reduce coercive practices in psychiatric wards (eAppendix 2 in Supplement 2). Evidence-based approaches were used to (1) identify a clinical problem related to coercion practices, (2) understand the problem using organizational information, (3) seek scientific evidence from the literature, and (4) consider stakeholder’s views (patients, nurses, and family members). An evidence-based pathway (eTable 1 in Supplement 2) was used to support changes in practices.^12^ The 8 months of education for staff (May 1 to December 31, 2016), included lectures, seminars, workshops, and side visits, was followed by a 10-month (January 1 to October 31, 2017) maintenance period to encourage and monitor ward activities. The usual practice wards continued with their normal activities.

### Outcomes

#### Primary Outcome

The primary outcome was the occurrence of patient seclusion events on a ward. The baseline data (January 1 to December 31, 2015) were collected retrospectively from ward registers before the intervention. The follow-up data for January 1 to December 31, 2017, were collected from hospital registers in 2018.

#### Secondary Outcomes

Secondary organizational outcomes included the number of patients secluded, the length of each seclusion event, and the use of other coercive methods aggregated at the ward level based on register data. In addition, information about service use was collected (type of admission, length of stay [in days], and death).

Nurse outcomes were nurses’ team climate (Team Climate Inventory)^13^ assessed by individual respondents at 2 time points (March-April 2016 and October 2017). Information about nurses’ turnover (resignations and filled positions) was based on registers at baseline and follow-up.

**Figure 1.**
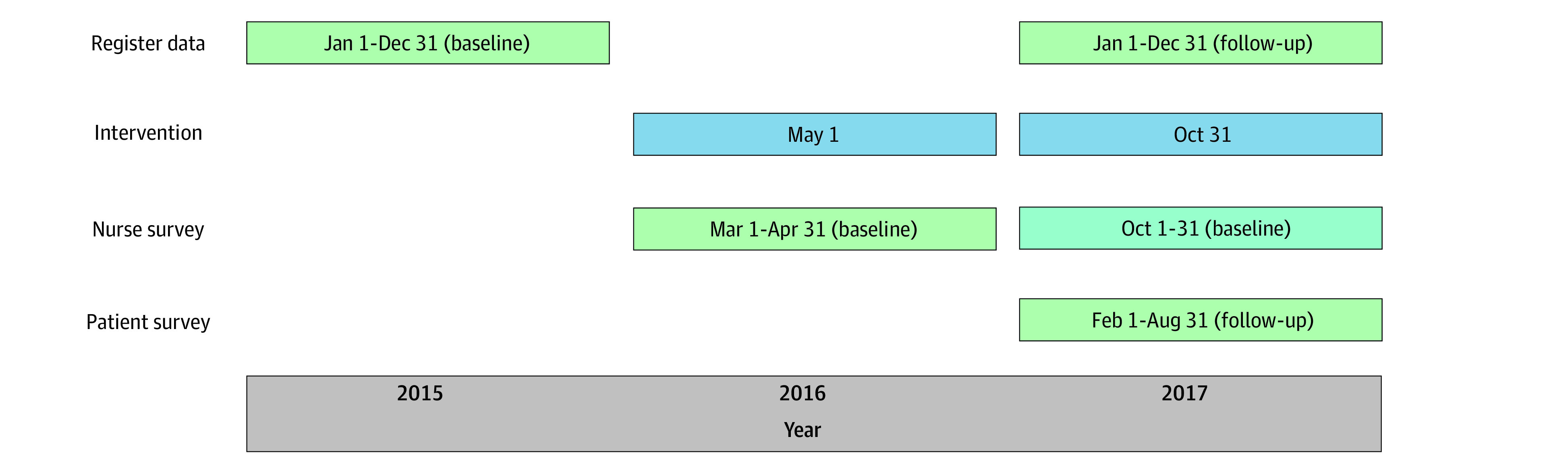
Timeline of Intervention and Outcome Data Collection

Patient outcome data were collected at the end of the study period for a 7-month period to avoid extra burden being put on nurses (February 1 to August 31, 2017). First, nurses assessed each patient’s overall functioning using the Global Assessment Scale (GAS).^14^ Second, patients joined a survey to assess their treatment satisfaction (8-item Client Satisfaction Questionnaire [CSQ-8]^15^) and quality of life (Quality of Life, Enjoyment, and Satisfaction Questionnaire [Q-LES-Q]^16^). A timeline of the intervention and the outcome data collection is presented in Figure 1. Adverse events were documented throughout the trial.

### Feasibility and Fidelity of the Intervention

Patients’ willingness to join the study and their awareness of the ongoing intervention were assessed, and opinions about the changes on the ward were explored.^12^ The degree of execution of the intervention on each ward was evaluated by 2 researchers (T.L. and M.A.). Ward integration into the intervention was assessed based on the number of wards that dropped out of the study. Intervention maintenance was monitored through monthly monitoring calls.

Fidelity assessment comprised 7 stages: acceptance, applicability, availability, ability, action on, agreement on, and adherence to the intervention.^17^ The percentage of aims that were fulfilled was assessed; each completed item received a score of 0 to 1.00, with 0 indicating low fidelity and 1.00 indicating high fidelity, giving a total percentage of 0% to 100%.^12^ Patients’ and relatives’ perceptions of the treatment environment collected in interviews are reported in a separate article.^18^

### Statistical Analysis

The sample size was calculated based on the design effect of the hospital numbers required for the analysis in a cluster randomized clinical trial (see trial protocol in Supplement 1).^11^ We assumed 7 clusters (hospitals) with 265 people in each intervention group would provide 80% power to detect a difference of 0.04 in the primary outcome (occurrence rate of seclusion at the ward level) between the 2 arms, with 0.11 in the usual practice group and 0.07 in the intervention group initially. A 2-sided *z* test (unpooled) with an overall significance level of *P* < .05 was used. Using hospital registers, we assumed the sample size for the total population admitted to the study wards in 1 year would be 3710. With a loss of 20% of patients in the local care registers, 4454 patients on the randomized wards were required. For the patient survey, assuming a 50% response rate, of a possible 3710 participants, we anticipated at least 928 completed patient questionnaires. The sample size calculation for the primary outcome at patient level was adjusted based on the number of admitted patients for intrahospital wards with a correlation of 0.005.

The characteristics of the wards and respondents (patients and staff members) were summarized with descriptive statistics (mean [SD] or numbers [percentages]) for each treatment group. All organizational outcomes were managed at the ward level because the data were not based on individual patients. For the primary outcome, the total number of secluded patients per ward during the baseline and follow-up periods were counted. A Poisson regression model with random effects for hospital was used to estimate the relative risk of seclusion events per ward, adjusting for mean age and sex (on ward level) at baseline in 2015 and at the end of the trial in 2017, using the total number of patients on each ward as an offset. The differences in the change of outcomes between the groups over time were tested with a group × year interaction effect. The same Poisson model was used for the secondary outcomes (total number of patients undergoing seclusion, limb restraint, forced injection, and physical restraint). Length per event and length of stay were analyzed using linear mixed model with random intercept for hospital. Logistic regression was used to analyze deaths, including a random intercept for hospital effect. The Hodges-Lehmann estimate with a 95% CI for the median difference between groups was calculated. Nurses’ team climate outcomes and patients’ outcomes (GAS, CSQ-8, and Q-LES-Q) were analyzed with a hierarchical linear model (participants nested within hospitals). Given the extent of the intracluster correlation on the power of the study, the intracluster correlation coefficient for each outcome analyzed was provided to assess the magnitude of the clustering for each outcome. Missing data were accommodated in the hierarchical model via random effects. Sensitivity analyses were conducted on wards in which participation was low to evaluate whether intervention fidelity has any effect to the results of the primary outcome. Data analysis was conducted on October 27, 2021. Results are expressed using rate ratios, odds ratios, or least-squares mean differences with corresponding 95% CIs, depending on the type of outcome. Statistical analyses were performed with SAS System for Windows, version 9.4 (SAS Institute Inc) and SPSS, versions 25.0 to 27.0 (IBM Corp). Because no adjustments for multiplicity of testing were made, only 95% CIs are reported for the secondary outcomes.

## Results

**Figure 2.**
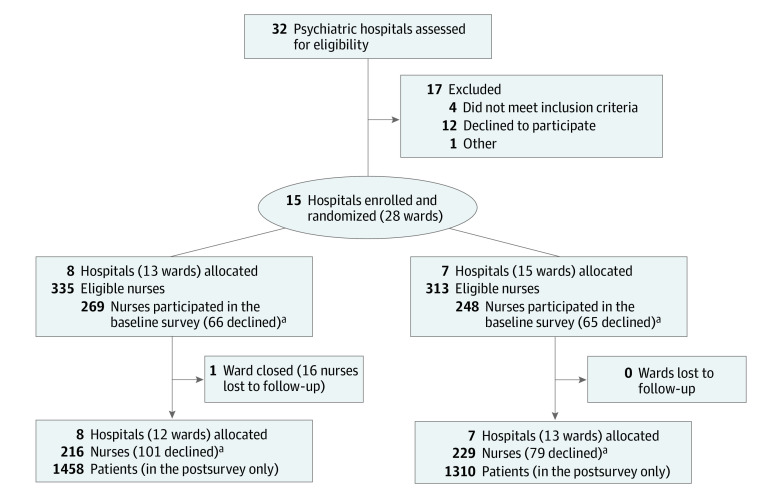
Trial Flow Diagram ^a^The survey was conducted on the ward level.

Of 28 psychiatric hospital wards screened (437 beds and 648 nurses), 27 wards completed the study. Eight wards were randomized to the intervention group (13 wards, 335 nurses, and 238 hospital beds) and 7 to the usual practice group (15 wards, 313 nurses, and 235 hospital beds). One ward was closed during the study period, and 16 nurses were lost to follow-up (Figure 2). A total of 8349 patients were were receiving care in the study wards, with 53% male patients and a mean (SD) age of 40.6 (5.7) years (Table 1). The overall number of seclusions was 1209 (14.5%) in 2015 and 1349 (16.5%) in 2017.

**Table 1.** Key Characteristics of the Psychiatric Hospitals, Nurses, and Patients^a^

Characteristic	Intervention (13 wards)^b^	Usual care (15 wards)	Total No.
**Unit characteristics at baseline (2015)**			
No. of hospital beds	238	235	473
No. of nurses	335	313	648
No. of patients on the study wards	4163	4186	8349
Bed-patient ratio on the ward, mean (SD)	0.1 (0.1)	0.1 (0.1)	0.1 (0.1)
Length of stay, mean (SD), d	23.2 (20.4)	31.8 (16.7)	27.8 (18.7)
Patient sex, %^c^			
Male	49	56	53
Female	51	44	47
Patient age, mean (SD), y	41.5 (6.5)	40.0 (5.1)	40.6 (5.7)
Involuntary admissions^d^	1576/4163 (37.9)	1766/4186 (42.2)	3342/8349 (40.0)
Patients involuntarily admitted	1146/4163 (27.5)	1488/4186 (35.5)	2634/8349 (31.5)
No. of nursing vacancies	311	309	620
**Nurse characteristics in survey**			
Age, mean (SD), y	41.8 (10.5)	42.7 (11.3)	42.2 (10.9)
No. of nurses who responded to the survey	269	247	516
Sex			
Male	129/269 (48.0)	117/248 (47.2)	246/517 (47.6)
Female	140/269 (52.0)	131/248 (52.8)	271/517 (52.4)
Work position			
Mental health, practical, or assistant nurse	67/264 (25.4)	77/244 (31.6)	144/508 (28.3)
Registered or specialized nurse	187/264 (70.8)	154/244 (63.1)	341/508 (67.1)
Head nurse	10/264 (3.8)	13/244 (5.3)	23/508 (4.5)
**Patient characteristics in survey**			
No. of patients who responded to the survey	1458	1310	2768
Age, mean (SD), y	40.1 (15.9)	36.5 (13.9)	38.5 (15.5)
No. of patients who responded to question about age	1410	1248	2658
Sex			
Male	671/1409 (47.6)	656/1244 (52.7)	1327/2653 (50.0)
Female	738/1409 (52.4)	588/1244 (47.3)	1326/2653 (50.0)
Marital status			
Single	787/1400 (56.2)	777/1241 (62.6)	1564/2641 (59.2)
Cohabiting or married	327/1400 (23.4)	265/1241 (21.4)	592/2641 (22.4)
Divorced	244/1400 (17.4)	181/1241 (14.6)	425/2641 (16.1)
Widowed	42/1400 (3.0)	18/1241 (1.5)	60/2641 (2.3)

^a^ Unless otherwise noted, all values are number of individuals/total number of respondents (percentage) for each category.

^b^ A total of 12 wards from 2016 onward.

^c^ This information is available only as percentages because not all wards were able to provide this information at a patient level.

^d^ Sample size is all patients because the number of treatment periods was not available.

In 2015, characteristics associated with the enrolled hospitals were balanced between treatment arms, with the exception of the number of hospital beds and involuntary admissions. The most common diagnoses based on the *International Statistical Classification of Diseases and Related Health Problems, Tenth Revision* (*ICD-10*) were schizophrenia, schizotypal and delusional disorders (codes F20-F29), mood (affective) disorders (codes F30-F39), neurotic, stress-related, and somatoform disorders (codes F40-F48), and factors influencing health status and contact with health services (codes Z00-Z99). Patient and nurse characteristics did not differ between the 2 groups, except for nurses’ regular working hours (Table 1). A detailed description of the organizations, nurses, and patients is available in eTable 2 in Supplement 2.

### Primary Outcome

The seclusion rate did not differ between the intervention group (629 of 4163 patients [15.1%]) and the usual practice group (580 of 4186 patients [13.9%]) in the baseline period in 2015 (adjusted rate ratio [ARR], 0.86; 95% CI, 0.40-1.82) (Table 2). In 2017, the occurrence rate of patient seclusion in the intervention group was 14.3% compared with 18.7% in the usual practice group (ARR, 0.66; 95% CI, 0.31-1.41). Over time, the intervention group achieved a small reduction in seclusion rate by 5.3% contrasted to an increase of 34.7% in the usual practice group (*P* = .003) (Table 2). The hospital-specific random-effects estimates with 95% CIs for the occurrence of seclusion (primary outcome) are described in the eFigure in Supplement 2.

**Table 2.** Organizational Outcomes Nested in Hospitals

Outcome	Baseline, year 2015		RR (95% CI)	*P* value	Adjusted RR (95% CI)^a^	*P* value	Year 2017		RR (95% CI)	*P* value	Adjusted RR (95% CI)^a^	*P* value	*P *value for interaction group × year	*P *value for adjusted interaction group × year^a^
	Intervention	Control					Intervention	Control						
**Primary outcome**														
No. of seclusion events at ward level/total No. of patients (%)^b^	629/4163 (15.1)	580/4186 (13.9)	0.93 (0.41 to 2.11)	.86	0.86 (0.40 to 1.82)	.68	585/4089 (14.3)	764/4092 (18.7)	0.72 (0.32 to 1.63)	.42	0.66 (0.31 to 1.41)	.27	.003	.003
**Secondary outcomes**														
No. of secluded patients at ward level/total No. of patients (%)	342/4163 (8.2)	354/4186 (8.5)	0.84 (0.44 to 1.61)	.59	0.83 (0.43 to 1.58)	.55	437/4089 (10.7)	512/4092 (12.5)	0.76 (0.40 to 1.46)	.41	0.75 (0.39 to 1.42)	.36	.37	.33
Length per seclusion event on ward level, geometric mean, min	1378	1614	−0.14 (−0.69 to 0.40)^c^	.60	−0.28 (−0.86 to 0.30)^c^	.33	1762	1614	0.16 (−0.39 to 0.71)^c^	.56	0.02 (−0.56 to 0.60)^c^	.94	.21	.19
No. of limb restraint events/total No. of patients (%)	360/4163 (8.7)	226/4186 (5.4)	2.36 (0.82 to 6.76)	.11	1.51 (0.45 to 5.14)	.50	353/4089 (8.6)	300/4092 (7.3)	1.39 (0.49 to 3.98)	.53	0.92 (0.27 to 3.11)	.89	<.001	.001
No. of patients on whom limb restraints were used/total No. of patients (%)	172/4163 (4.1)	126/4186 (3.0)	2.15 (0.77 to 6.01)	.14	1.65 (0.53 to 5.13)	.38	235/4089 (5.8)	202/4092 (5.0)	1.59 (0.57 to 4.41)	.36	1.23 (0.40 to 3.80)	.71	.06	.07
Length per limb restraint event, geometric mean, min	1345	851	0.84 (−0.19 to 1.86)^c^	.11	0.67 (−0.32 to 1.66)^c^	.18	911	924	0.42 (−0.62 to 1.46)^c^	.42	0.18 (−0.86 to 1.21)^c^	.73	.26	.19
No. of forced medication events/total No. of patients (%)	317/4163 (7.6)	414/4186 (9.9)	0.65 (0.31 to 1.38)	.26	0.61 (0.29 to 1.28)	.18	486/4089 (11.9)	481/4092 (11.8)	0.87 (0.41 to 1.83)	.71	0.81 (0.39 to 1.68)	.56	.007	.008
No. of patients injected/total No. of patients (%)	150/4163 (3.6)	295/4186 (7.1)	0.56 (0.26 to 1.18)	.12	5.55 (0.26 to 1.13)	.10	292/4089 (7.1)	289/4092 (7.1)	1.12 (0.53 to 2.36)	.76	1.09 (0.53 to 2.24)	.81	<.001	<.001
No. of physical restraint events/total No. of patients (%)	38/4163 (0.9)	27/4186 (0.7)	3.53 (0.63 to 19.74)	.15	4.52 (0.67 to 30.40)	.12	98/4089 (2.4)	29/4092 (0.7)	5.04 (0.94 to 26.96)	.06	6.25 (0.99 to 39.64)	.05	.29	.34
No. of patients physically restrained/total No. of patients (%)	23/4163 (0.6)	11/4186 (0.3)	2.70 (0.58 to 12.54)	.20	2.01 (0.43 to 9.48)	.37	70/4089 (1.7)	14/4092 (0.3)	4.74 (1.14 to 19.78)	.03	3.69 (0.89 to 15.25)	.07	.25	.22
Length per event, geometric mean, min	28	25	0.02 (−2.28 to 2.32)^c^	.98	0.74 (−1.51 to 3.00)^c^	.47	8	30	−1.33 (−3.52 to 0.86)^c^	.21	−0.69 (−2.82 to 1.45)^c^	.48	.16	.13
Length of stay per treatment period, geometric mean, d	16	26	−0.52 (−1.26 to 0.28)^c^	.17	−0.75 (−1.43 to −0.06)	.03	14	21	−0.42 (−1.17 to 0.33)^c^	.26	−0.66 (−1.35 to 0.03)^c^	.06	.82	.81
Death (yes)	5	1	5.26 (0.45 to 61.83)^d^	.18	3.34 (0.26 to 42.11)^d^	.34	4	1	4.59 (0.37 to 56.69)^d^	.23	3.31 (0.27 to 40.32)^d^	.34	.93	>.99

Abbreviation: RR, risk ratio.

^a^ Adjusted for mean age and sex (on ward level).

^b^ The total number of seclusion events on the ward is given because patient-level information for seclusions was not available.

^c^ Least-squares mean difference (95% CI); log-transformed values were used in statistical analysis and linear mixed model with random intercept for hospital.

^d^ Odds ratio (95% CI); logistic regression with random intercept for hospital.

### Secondary Outcomes

The proportion of patients secluded in the 2 groups was no different in 2015 (ARR, 0.83; 95% CI, 0.43-1.58) than in 2017 (ARR, 0.75; 95% CI, 0.39-1.42). Both groups showed a similar increase in the outcome. The seclusion duration was no different in 2015 (geometric mean, −0.28; 95% CI, −0.86 to 0.30) than in 2017 (geometric mean, 0.02; 95% CI, −0.56 to 0.60) and did not change over time. On the other hand, there was an increase from 226 events among 4186 patients (5.4%) in 2015 to 300 events among 4092 patients (7.3%) in 2017 in the occurrence of limb restraint per ward in the usual practice group compared with little change (360 events among 4163 patients [8.6%] in 2015 to 353 events among 4089 patients [8.6%] in 2017) in the intervention group (*P* < .001). However, no difference or change over time in the proportion of limb restraint or in the median length of limb restraint event was found between the 2 groups. The occurrence rate of forced injection in both groups did not differ at baseline (ARR, 4.52; 95% CI, 0.67-30.40) or in 2017 (ARR, 5.04; 95% CI, 0.94-27.00). However, the increase of 56.2% from 7.6% to 11.9% over time in the intervention group was significantly larger than the small increase of 7.7% in the usual practice group (*P* < .001). Following the same pattern, the proportion of patients who received a forced injection increased more in the intervention group than in the usual practice group, although the ARRs were not statistically significant for both 2015 (ARR, 0.55; 95% CI, 0.26-1.13) and 2017 (ARR, 1.09; 95% CI, 0.53-2.24). No differences were found between the 2 groups in the occurrence rate of physical restraint, proportion of patients physically restrained, or length of time per event (Table 2).

Nurse turnover rates on psychiatric wards appeared no different in the intervention vs the usual care group. Similarly, no intervention effects were found in the total measure or submeasures of the team climate (eTable 3 in Supplement 2). No differences in functional capacity (GAS), treatment satisfaction (CSQ-8), or quality of life (Q-LES-Q) were observed in patients (eTable 4 in Supplement 2).

### Intervention Feasibility and Fidelity

Of 36 study visits conducted by the research team, patients joined in 35 meetings (acceptability, 92% of a target of 95%). The educational intervention was easy to put into practice (92%), and fidelity was high (85%) (eTable 5 in Supplement 2). Although patients on all wards supported the proposed improvements, on 6 wards, patients were not fully aware of the ongoing improvements on the wards. Examples of specific activities conducted by nurses are described in eTable 6 in Supplement 2.

### Sensitivity Analysis

A sensitivity analysis was conducted by comparing the primary data at follow-up and the results on study wards where nurse and/or patient participation was low. The analysis showed similar results to those of the primary analysis after excluding the wards with a low participation rate (eTable 7 in Supplement 2), suggesting good fidelity of the trial.

### Adverse Events

Seven adverse events were reported: 2 severe life-threatening adverse events occurred (both not likely related to the intervention) and 5 mild events. The chief psychiatrist was consulted regarding the 2 severe cases, and no changes in the intervention were requested based on these cases.

## Discussion

Contrary to previous studies,^19,20,21^ in this cluster randomized clinical trial, although feasible, we found only weak evidence that the effect of the evidence-based educational intervention for nurses reduced the occurrence of seclusion events at the hospital level. The major difference from previous studies^19,20,21^ was that we used a tailored approach, which may not be strong enough to show positive effects on a hospital level. It is also unclear which aspects of the intervention were effective in reducing restriction practices. Indeed, there is still insufficient evidence on the most effective approaches to tailoring, including how decisions should be made on which determinants are most important and how interventions should be selected to account for the important determinants.^22^ Further research could seek a stronger and more direct estimate for causal inference on the subject.^7^ In addition, as shown in the intracluster correlation, the intervention might have been well implemented on some wards but ignored on other wards, which limits its robustness, perhaps depending on how nurse leaders in clusters supported the adaptation process. On the other hand, our sensitivity analysis based on the fidelity of the intervention showed no changes in the study results. Thus, our study supports the recent review by Geoffrion et al,^23^ which showed that education combined with training may not affect workplace aggression directed toward health care workers. More studies are therefore needed to better understand the mechanism of effective interventions to reduce coercive practices.

In our study, the use of forced injection and the number of patients injected increased at the same time that the occurrence of seclusion events decreased. On one hand, nurses might have preferred involuntary medication^24^ and replaced seclusion events with forced injections; nurses are eager to switch to less restrictive coercive methods.^25^ On the other hand, increase in forced injections can also be seen on the national level in Finnish national statistics,^26^ perhaps because of changes in the reporting system. Previously, forced injection was included as a coercive method, but the registration widened in 2016 to also capture forced medication.^27^

### Strengths and Limitations

The inclusion of psychiatric hospitals across diverse regions of Finland is a strength of this study. The intervention was based on real clinical problems, and the solutions were based on evidence. As already recommended,^23^ we examined the longer-term effect on institutional level using existing follow-up register data and used employee turnover as an outcome. We also used user-centered approaches and encouraged the involvement of family and patients.^7^ In general, our study can add new information to the current research context (eAppendix 3 in Supplement 2).

At the same time, as a practical trial, the study reflected current clinical problems. Because of the limitations in hospital registration systems, not all study wards were able to offer individual-level data on patient coercion. Therefore, our primary outcome data were analyzed at the ward level, which limited in-depth investigation on the effects of the intervention on the outcomes. Another explanation to our study findings might be underpowered study results, particularly underestimated random effects at the hospital level in the trial design. Any modest effects still need much bigger trials to confirm these outcomes at the individual level.

## Conclusions

The education intervention did not convincingly reduce coercive practice in this randomized clinical trial, which is the largest of its kind to our knowledge. The findings are still compatible with this intervention having an effect and changing practice toward what are seen as less restrictive and more humane measures. Considering all the available evidence, multicomponent educational interventions for nurses could reduce the use of seclusion rooms and the number of patient restrictions in psychiatric hospitals, although the use of forced medication may concurrently increase.

## Notes

Supplement 1.
Trial Protocol

Supplement 2. **eAppendix 1.** Differences Between the Protocol and Trial **eAppendix 2.** Curriculum of the Evidence-Based Training Program for Nurses (VIOLIN) **eTable 1.** Description of the Intervention Pathway **eTable 2.** Detailed Characteristics of the Psychiatric Hospital Units, Nurses, and Patients **eFigure.** The Hospital-Specific Random Effect Estimates With 95% Confidence Intervals for the Occurrence of Seclusion (Primary Outcome) **eTable 3.** Secondary Outcome Analysis of Nurse Variables **eTable 4.** Secondary Outcome Analysis of Patient Variables **eTable 5.** Intervention Fidelity (Glasziou and Haynes et al, 2005) **eTable 6.** Examples of Intervention Activities **eTable 7.** Sensitivity Analysis **eAppendix 3.** Evidence Existing Prior to This Study
eReferences

Supplement 3.
Data Sharing Statement
