# Supplementary material for: An Evidence-Based Educational Intervention for Reducing Coercive Measures in Psychiatric Hospitals: A Randomized Clinical Trial
Source: JAMA Netw Open. 2022 Aug 30;5(8):e2229076. doi: 10.1001/jamanetworkopen.2022.29076 (PMC9428738; doi:10.1001/jamanetworkopen.2022.29076)
Supplement: Supplement 2. — eAppendix 1. Differences Between the Protocol and Trial eAppendix 2. Curriculum of the Evidence-Based Training Program for Nurses (VIOLIN) eTable 1. Description of the Intervention Pathway eTable 2. Detailed Characteristics of the Psychiatric Hospital Units, Nurses, and Patients eFigure. The Hospital-Specific Random Effect Estimates With 95% Confidence Intervals for the Occurrence of Seclusion (Primary Outcome) eTable 3. Secondary Outcome Analysis of Nurse Variables eTable 4. Secondary Outcome Analysis of Patient Variables eTable 5. Intervention Fidelity (Glasziou and Haynes et al, 2005) eTable 6. Examples of Intervention Activities eTable 7. Sensitivity Analysis eAppendix 3. Evidence Existing Prior to This Study eReferences [file jamanetwopen-e2229076-s002.pdf]

# Supplementary Online Content

Välimäki M, Lantta T, Anttila M, Vahlberg T, Normand SL, Yang M. An evidence-based educational intervention for reducing coercive measures in psychiatric hospitals: a randomized clinical trial. *JAMA Netw Open*. 2022;5(8):e2229076. doi:10.1001/jamanetworkopen.2022.29076

**eAppendix 1.** Differences Between the Protocol and Trial

**eAppendix 2.** Curriculum of the Evidence-Based Training Program for Nurses (VIOLIN)

**eTable 1.** Description of the Intervention Pathway

**eTable 2.** Detailed Characteristics of the Psychiatric Hospital Units, Nurses, and Patients

**eFigure.** The Hospital-Specific Random Effect Estimates With 95% Confidence Intervals for the Occurrence of Seclusion (Primary Outcome)

**eTable 3.** Secondary Outcome Analysis of Nurse Variables

**eTable 4.** Secondary Outcome Analysis of Patient Variables

**eTable 5.** Intervention Fidelity (Glasziou and Haynes et al, 2005)

**eTable 6.** Examples of Intervention Activities

**eTable 7.** Sensitivity Analysis

**eAppendix 3.** Evidence Existing Prior to This Study

**eReferences**

**This supplementary material has been provided by the authors to give readers additional information about their work.**

© 2022 Välimäki M et al. *JAMA Network Open.*

## eAppendix 1. Differences between the protocol and trial

In the protocol, the primary outcome was incidence of seclusion room use by individual patients. In the trial, however, we were not able to calculate incidence rate at the patient level due to a lack of information on individual patients for each treatment period. Hence, we evaluated the occurrence rate of seclusion use on ward level. Our sensitivity analysis described in the Results suggest that the key finding in the primary outcome was robust and not affected by the change in data analysis plan.

We would have preferred to analyse the data from three time points (2015, 2016, 2017). However, the data were analysed at two time points only (baseline vs. post-allocation) because the intervention periods partially overlapped in year 2016.

Another set of analysis was conducted to take into account a possible hospital effect on study results.

**eAppendix 2. Curriculum of the Evidence-Based Training Program for Nurses (VIOLIN)**

**Aim**

Students are able to use knowledge and skills to implement evidence-based practice in psychiatric inpatient care.

**Sub-goals and learning outcomes**

1. Students are aware of the evidence-based methods for reducing violence and coercion in psychiatric care
2. Students are aware of the different implementing strategies and outcomes for implementation of evidence-based practice in mental health area
3. Students are able to use evidence-based decision-making to facilitate changes in clinical practice.
4. Students are able to identify and analyse clinical situations and the steps needed toward evidence-based practice
5. Students understand and are able to great positive culture for evidence-based practice.

**Content**

Evidence-based methods for reducing violence and coercion in psychiatric care
Evidence-based decision-making
Different implementing strategies
Identifying and analyzing needs for changes in clinical practice
Facilitating changes in clinical practice
Outcome measures assessing change in clinical practice
Creating positive culture for evidence-based practice

**Learning methods**

| | |
|---|---|
| Lecturing | 16 h |
| Small groups | 8 h |
| Group assignments | 38 h |
| Group interview participation | 2 h |
| Workshops (3) | 24 h |
| Monthly maintaining calls with facilitators | 2h |

**Required learning activities**

Analyzing violent events on the wards
SWOT analysis related to implementing changes
Planning the changes on the wards based on detected needs for changes
Participation in workshops with lectures
Participating in site visits at hospital
Seminars
Participation in group interviews

**Study material**

Lecturing material
Assessment of the organizational situation

**Assessment**
Implementation of planned changes were assessed by researchers on a ward level

**Length**
Totally 18 months

- 8 months training period
- 10 month maintaining period

**Further information**
The program material is offered in Finnish only.

**Responsible persons**
Professor Maritta Valimaki, mava@utu.fi

**Traineers**
Tella Lantta, tejela@utu.fi
Minna Anttila, minant@utu.fi

© 2022 Välimäki M et al. *JAMA Network Open.*

**eTable 1. Description of the evidence-based intervention pathway**

| Phases | What happened in the intervention? |
| --- | --- |
| **Phase 1**<br>***Acceptance*** | • To ensure acceptance of the changes, possible problems in current treatment practices in each ward identified by analysing the current ward ‘rules’; the quality of the service facilities assessed from the point of views of patients, families, and staff members.<br>• Opinions about the possible changes discussed and the quality gaps in services identified.<br>• Areas to be developed were identified in consultation with staff members in two educational seminars (8/2016, 11/2016)<br>• The strengths, weaknesses, opportunities, and threats (SWOT) related to future changes identified. |
| **Phase 2**<br>***Applicability*** | • The facilitators visited the intervention wards two times times during active education phase (5-6/2016, 12/2016-1/2017) and later in maintaining phase (10/2017) to support identification of ward-specific quality gaps, and discussing with ward managers, patients, relatives and staff members at local meetings.<br>• Barriers and facilitating factors for change identified.<br>• Decisions made regarding what development activities to be done and how |
| **Phase 3**<br>***Available*** | • The evidence-based part supported by learning material to ensure staff’s competence to make managed changes regarding treatment practices. |
| **Phase 4**<br>***Able*** | • The facilitators made monthly calls to on each ward to prompt, encourage and monitor new treatment practices (1-9/2017).<br>• Ongoing identification of areas to be developed were discussed in one seminar (5/2017).<br>• Continuity of identification of ward-specific quality gaps, and discussing with ward managers, patients, relatives and staff members at local meetings was supported in seminar (10/2017)<br>• Possible problems in changing practices shared, and solutions further explored.<br>• Project leaflets were distributed to encourage staff members and disseminate progress and achievement of the study. |
| **Phase 5**<br>***Acted on*** | • The facilitators offered hands-on support to study ward to ensure that wards were acting based on the specific implementation plan (problems identified on the ward, a new solution found for their problems, maintaining new practices).<br>• Each ward monitored in their progress using a specific monitoring form as evidence of the changes in clinical practice. |
| **Phase 6**<br>***Agreed on*** | • As evidence of changes in daily practices, the content of the monitoring forms and local house rules re-analysed.<br>• Changes in treatment practices were recorded by comparing the situation at the beginning and at the end of the project. |
| **Phase 7**<br>***Adhered*** | • Staff adherence of the educational process was evaluated by assessing how specific intervention fidelity criteria had been fulfilled. |

© 2022 Välimäki M et al. *JAMA Network Open.*

**eTable 2. Detailed characteristics of the psychiatric hospital units, nurses, and patients**

| | **Intervention (n = 13[a])** | **Care as Usual (n = 15)** | **Total** |
|---|---|---|---|
| **Unit characteristics, year 2015** | | | |
| Number of hospital beds | 238 | 235 | 473 |
| Number of nurses | 335 | 313 | 648 |
| Number of patients[b] | 4163 | 4186 | 8349 |
| Bed/patient ratio on the ward[c] | 0.1 (0.1) | 0.1 (0.1) | 0.1 (0.1) |
| Length of stay, mean (SD) | 23.2 (20.4) | 31.8 (16.7) | 27.8 (18.7) |
| Patient gender % | | | |
| Male | 49 | 56 | 53 |
| Female | 51 | 44 | 47 |
| Patient age mean (SD) | 41.5 (6.5) | 40.0 (5.1) | 40.6 (5.7) |
| Three most common diagnoses based on ICD-10 | 1. F20-F29d[f]<br>2. F30-F39[g]<br>3. F40-F48[h] | 1. F20-F29[f]<br>2. F30-F39[g]<br>3. Z00-Z99[i] | 1. F20-F29[f]<br>2. F30-F39[g]<br>3. Z00-Z99[i] |
| Number of involuntary admissions: n/N[d] (%) | 1576/4163 (38) | 1766/4186 (42) | 3342 |
| Patients involuntarily admitted: n/N (%) | 1146/4163 (28) | 1488/4186 (36) | 2634 |
| Number of nursing vacancies | 311 | 309 | 620 |
| **Nurse characteristics in survey[e]** | | | |
| Age (years): mean (SD), N | 41.8 (10.5); N=269 | 42.7 (11.3); N=247 | 42.2 (10.9); N=516 |
| **Gender: n/N (%)** | | | |
| Male | 129/269 (48) | 117/248 (47) | 246/517 (48) |
| Female | 140/269 (52) | 131/248 (53) | 271/517 (52) |
| **Work position** n/N (%) | | | |
| Mental health, practical or assistant nurse | 67/264 (25) | 77/244 (32) | 144/508 (28) |
| Registered or specialised nurse | 187/264 (71) | 154/244 (63) | 341/508 (67) |
| Head nurse | 10/264 (4) | 13/244 (5) | 23/508 (5) |
| **Employment data** | | | |
| Permanent | 204/266 (77) | 196/245 (80) | 400/511 (78) |
| Temporary or other | 62/266 (23) | 49/245(20) | 111/511 (22) |
| Years working in this hospital: mean (SD) | 12.2 (9.4); N=268 | 12.2 (10.5); N=246 | 12.2 (9.9); N=514 |
| Years working in current position: Mean (SD) | 8.6 (8.6); N=266 | 8.5 (8.8); N=248 | 8.6 (8.5); N=514 |
| Years working in mental health field: mean (SD) | 13.8 (9.6); N=268 | 14.8 (10.8); N=248 | 14.3 (10.2); N=516 |

© 2022 Välimäki M et al. *JAMA Network Open.*

| | | | |
|---|---|---|---|
| **Nurses' regular working hours** | | | |
| Regular day shift: n/N (%) | 15/268 (6) | 19/245 (8) | 34/513 (7) |
| Shift work (2 shifts): n/N (%) | 44/268 (16) | 30/245 (12) | 74/513 (14) |
| Shift work (3 shifts): n/N (%) | 189/268 (71) | 194/245 (79) | 383/513 (75) |
| Regular night shift or other: n/N (%) | 20/268 (7) | 2/245 (1) | 22/513 (4) |
| **Patient characteristics in survey** | **Intervention (n=1458)** | **Care as usual (n=1310)** | Total |
| **Age in years: mean (SD)** | 40.1 (15.9); N=1410 | 36.5 (13.9); N=1248 | 38.5 (15.5); N=2658 |
| **Gender: n/N (%)** | | | |
| Male | 671/1409 (48) | 656/1244 (53) | 1,327/2653 (50) |
| Female | 738/1409 (52) | 588/1244 (47) | 1,326/2,653 (50) |
| **Marital status: n/N (%)** | | | |
| Single | 787/1400 (56) | 777/1241 (63) | 1564/2641 (59) |
| Cohabiting/married | 327/1400 (23) | 265/1241 (21) | 592/2641 (23) |
| Divorced | 244/1400 (18) | 181/1241 (15) | 425/2641 (16) |
| Widowed | 42/1400 (3) | 18/1241 (1) | 60/2641 (2) |
| **Education level: n/N (%)** | | | |
| No formal education | 61/1393 (4) | 60/1255 (5) | 121/2618 (5) |
| Middle or comprehensive school | 371/1393 (27) | 361/1255 (29) | 732/2618 (28) |
| High school | 227/1393 (16) | 202/1255 (17) | 429/2618 (16) |
| Vocational school or courses | 537/1393 (38) | 419/1255 (34) | 956/2618 (36) |
| Lower secondary education | 121/1393 (9) | 111/1255 (9) | 232/2618 (9) |
| Higher secondary education | 67/1393 (5) | 62/1255 (5) | 129/2618 (5) |
| Doctoral degree | 9/1393 (1) | 10/1255 (1) | 19/2618 (1) |
| **Housing: n/N (%)** | | | |
| With parents | 119/1363 (9) | 103/1183 (8) | 222/2546 (9) |
| With spouse/partner/child (no parents) | 387/1363 (28) | 305/1183 (26) | 692/2546 (27) |
| Alone, roommates, other relatives | 680/1363 (50) | 592/1183 (50) | 1272/2546 (50) |
| Supported living | 171/1363 (13) | 177/1183 (15) | 348/2546 (13) |
| Homeless | 6/1363 (0) | 6/1183 (1) | 12/2546 (1) |
| **Employment status: n/N (%)** | | | |
| Unemployed | 305/1413 (22) | 369/1251 (30) | 674/2664 (25) |
| Employed | 268/1413 (19) | 199/1251 (16) | 467/2664 (18) |
| Student | 121/1413 (9) | 102/1251 (8) | 223/2664 (8) |
| Self-employed person | 14/1413 (1) | 15/1251 (1) | 29/2664 (1) |
| Retired | 594/1413 (42) | 448/1251 (36) | 1042/2664 (39) |
| Other, undetermined | 111/1413 (8) | 118/1251 (9) | 229/2664 (9) |

© 2022 Välimäki M et al. *JAMA Network Open.*

| | | | |
|---|---|---|---|
| **Number of psychiatric treatment periods: n/N (%)** | | | |
| None | 6/1281 (1) | 8/1128 (1) | 14/2409 (1) |
| 1 | 299/1281 (23) | 266/1128 (23) | 565/2409 (23) |
| 2–4 | 477/1281 (37) | 459/1128 (41) | 936/2409 (39) |
| 5–8 | 218/1281 (17) | 184/1128 (16) | 402/2409 (17) |
| 9 or more | 191/1281 (15) | 147/1128 (13) | 338/2409 (14) |
| Many/undefined number | 90/1281 (7) | 64/1128 (6) | 154/2409 (6) |
| **Age of first contact with services: n/N (%)** | | | |
| 0–14 years | 135/1272 (11) | 153/1121 (13) | 288/2393 (12) |
| 15–24 years | 588/1272 (46) | 550/1121 (49) | 1138/2393 (48) |
| 25–44 years | 396/1272 (31) | 332/1121 (30) | 728/2393 (30) |
| 45–64 years | 124/1272 (10) | 76/1121 (7) | 200/2393 (8) |
| 65 years or older | 29/1272 (2) | 10/1121 (1) | 39/2393 (2) |

[a]12 wards 2016 onwards;
[b]Total number of patients on the study wards.
[c]Ratio of beds and total number of patients in each ward/year.
[d]N=all patients, number of treatment periods not available.
[e]Number of respondents varies in each item.
[f]F20-F29 Schizophrenia, schizotypal and delusional disorders.
[g]F30-F39 Mood [affective] disorders.
[h]F40-F48 Neurotic, stress-related and somatoform disorders.
[i]Z00-Z99 Factors influencing health status and contact with health services.

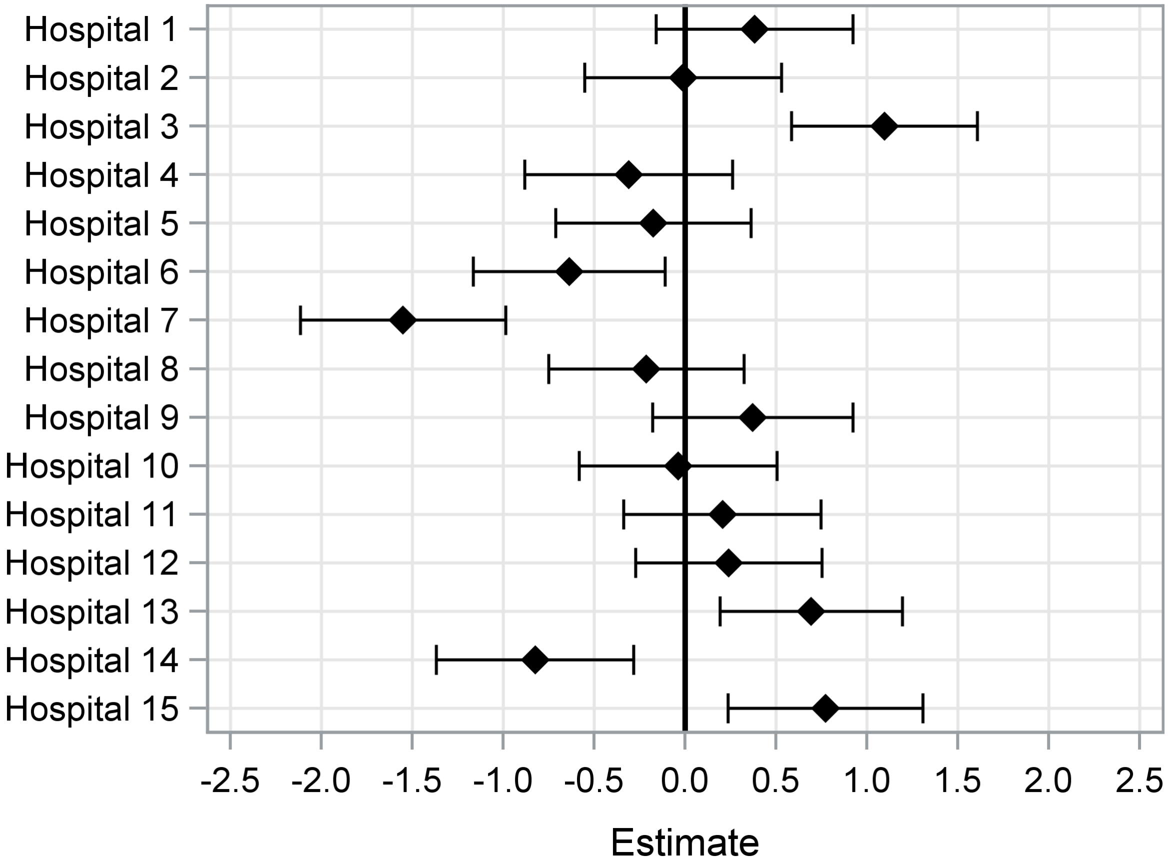


**eFigure. The hospital-specific random effect estimates with 95% confidence intervals for the occurrence of seclusion (primary outcome)**

**eTable 3. Secondary outcome analysis of nurse variables**

| Variables | Intervention (N=269, survey) | Usual practice (N=248, survey) | | Intervention (N=216, survey) | Usual practice (N=229, survey) | | |
|---|---|---|---|---|---|---|---|
| | Baseline, 2016 (N = 517) | | LSM (95% CI) | Follow-up, 2017 (N = 439) | | LSM (95% CI) | ICC |
| Resignations, mean (SD)/median | 0.46 (0.66)/0.00 | 1 (2.39)/0.00 | 0.00 (0.00; .000)[a] | 0·33 (0·49)/0 | 1·07 (1·53)/1.00 | 0.00 (-1.00; 0.00)[a] | |
| **Team climate (total),** mean (SD) | 4.35 (0.69) | 4.19 (0.66) | 0.14 (-0.09; 0.37) | 4.37 (0.69) | 4.31 (0.63) | 0.01 (-0.23; 0.24) | 0.08 |
| Participative safety | 3.82 (0.58) | 3.68 (0.58) | 0.13 (-0.04; 0.29) | 3.88 (0.61) | 3.76 (0.54) | 0.08 (-0.09; 0.25) | 0.05 |
| Support for innovation | 3.37 (0.68) | 3.19 (0.68 | 0.16 (-0.08; 0.39) | 3.39 (0.70) | 3.32 (0.65) | 0.03 (-0.21; 0.27) | 0.08 |
| Vision | 5.26 (0.91) | 5.12 (0.93) | 0.12 (-0.16; 0.40) | 5.24 (0.95) | 5.24 (0.85) | -0.05 (-0.34; 0.24) | 0.06 |
| Task orientation | 4.94 (1.01) | 4.75 (0.94) | 0.16 (-.14;0.45) | 4.96 (0.99) | 4.94 (0.88) | -0.03 (-0.34; 0.27) | 0.06 |

[a]Hodges-Lehmann estimate (95% confidence interval) for median difference between groups
ICC = Intraclass correlation coefficient (participants nested on hospital level)
LSM = Least square mean difference estimated from hierarchical linear model (participants nested within hospitals)
CI = Confidence interval

© 2022 Välimäki M et al. *JAMA Network Open.*

**eTable 4. Secondary outcome analysis of patient variables**

| | **Intervention wards (N = 12)** | **Usual practice wards (N = 15)** | **LSM (95% CI)** | **ICC** |
|---|---|---|---|---|
| **PATIENT OUTCOMES** | | | | |
| [a]Functional capacity (GAS), mean (SD) | 49.15 (13.11); n=2384 | 47.25 (13.55); n=2148 | 4.27 (-1.67; 10.21)[c] | 0.18 |
| [b]Patient treatment satisfaction (CSQ-8) | 24.77 (5.04); n=1403 | 24.51 (4.99); n=1228 | 0.29 (-0.70; 1.29)[d] | 0.03 |
| Quality of life (Q-LES-Q) | 45.36 (10.95); n=1366 | 46.72 (11.48); n=1202 | -1.98 (-4.06; 0.10)[d] | 0.03 |

[a]Assessed by nurses during patient discharge process as routine data collection
[b]The data collected by patients' survey
[c]Hierarchical linear model (participants nested within hospitals) (GAS)
[d]Hierarchical linear model (participants nested within hospitals), adjusted for age, gender, occupational status, and age at first contact with psychiatric services
LSM = Least square mean difference estimated from hierarchical linear model (participants nested within hospitals)
CI = Confidence interval
ICC = Intraclass correlation coefficient (participants nested on hospital level)

© 2022 Välimäki M et al. *JAMA Network Open.*

**eTable 5. Intervention fidelity (Glasziou and Haynes et al, 2005)***

| Implementation stage in interventional wards | Achieved assessment (fidelity criteria) | Achieved % / (Aim %) |
|---|---|---|
| **Acceptance**<br>Two one-day workshops for contact persons to share the results of the need analysis and design a preliminary action plan.<br>A local ward meeting between staff organised by contact person.<br><br>'House rules' collected and analysed (at baseline) | At least one contact person attended each workshop<br><br>The local ward meetings organised and documented on each ward<br><br>'House rules' analysed | 1st 100/(80)<br>2nd 85/(80)<br><br><br>100/(100)<br><br>100/(100) |
| **Applicable**<br>A series of local meetings with staff, patients, and relatives was organised; development areas and development steps identified; SWOT analysis conducted | The first visit on each ward by researchers<br><br>Staff from each ward participated in the visit | 100/(100)<br><br>1st 58/(50) |
| **Available**<br>An information package of the intervention available for staff | Action Plan developed for each ward; content of the information package shared with staff | 100/(100) |
| **Able**<br>Monthly monitoring calls to prompt and encouraged changes | Contact persons were called to get a report of the progress and possible harms (12 calls or email/12 months) | 100/(100) |
| **Acted on**<br>Visits to each ward organised by researchers to give hands-on support (workshops and on-site meetings, and regular call to discuss the current situation; | 2nd visit organised by researchers. At least 50% of the staff on the ward attended the visits<br>3rd workshop organised by researchers; at least one person from each unit attended (Interim Evaluation) | 64/(50)<br><br>3rd 92/(95) |

© 2022 Välimäki M et al. *JAMA Network Open.*

| | | |
|---|---|---|
| targeted information package based on the literature search) how to make changes in treatment practices; Action Plan revised (if needed); one-day 3rd workshop organised for interim evaluation | | |
| **Agreed on**<br>'House rules' recollected and analysed; differences in previous and current actions identified | Patient coercive methods and house rules analysed | 100/(100) |
| **Adhered to**<br>Daily practices monitored; final workshop organised | Daily practices monitored; outcomes of the intervention evaluated based on the number of nurses attended in a meeting/ward.<br>At least one contact person from each ward attended in the 4th (final) workshop. | 3rd 60/(50)<br><br>92/(95) |

*Modified based on Killapsy 2015[12]

© 2022 Välimäki M et al. *JAMA Network Open.*

| **eTable 6. Examples of intervention activities** | | | | | | |
|---|---|---|---|---|---|---|
| **Most commonly described problems/needs at the wards** | **Ward**<br><br>**N** | **What has been done to solve the problem?** | **Possible risks for change** | **Possible risks of the intervention** | **Expected and experienced results** | **Realized risk** |
| **Patients do not feel safe in the ward**<br>- Feelings of unsafety in patients<br>- Nurses not present and do not spend time with the patients i.e. spend too much time in the nurses station<br>-Problems in improving safety and predicting issues in the ward<br>- Conflicts between the patients | 7 | - Nurses are always present and spend time in common areas on the ward the, e.g. at least one hour /shift per nurse. They aim to make the atmosphere safe and they can answer to the needs of the patients easier. | -Some nurses and patients do not see nurses presence on the ward important. Nurses resistance to change.<br>- Amount of paper work required from the staff or sudden situations can decrease nurses' presence<br>- The presence of nurses is not evaluated often enough<br>-Patients may be in state of confusion, agitated, have a flow of words and have disturbances in the interaction. Thus providing presence to patients may not be always rewarding for the staff and | - Even thought a nurse is present on the ward, some patients still might come to nurses station for requests<br>- Providing presence can be exhausting to the nurses, the fear of overburden, because patients are in poor shape and increase of psychological strain.<br>- Presence can be used to avoid other work duties. | -Nurses have recognized the benefits of being available and visible, patients feeling safer and anxiety being reduced, more welcome and more relaxed atmosphere in common areas<br>- Nurses in the nurses station can focus more on their work.<br>-Patients get more time from the nurses, improves patient being aware what is going on in the ward (e.g about daily activities).<br>- The staff can react to challenging situations faster. Conflicts between patients can be prevented.<br>- Reducing the use of coercive measures.<br>-The ward is calmer | No risks identified |

© 2022 Välimäki M et al. *JAMA Network Open.*

| | | | | | | |
|---|---|---|---|---|---|---|
| | | | motivating the staff for this task may be difficult. | | | |
| **Pointless restrictions in care**<br>-Old rules are not up-to-date and are more restrictive than guiding in their nature.<br>-Patients are not aware of all the ward rules (e.g. is it allowed to go to other patients' rooms) | 5 | -Updating the ward rules (ward or hospital-level)<br>- Making the rules visible and more understandable to the patients. | -The staff not engaged with updated rules<br>- Patients' do not understand expectations. | -After updating the rules, the patients are not aware what is expected from them | - Everyone have the same understanding about updated ward rules and safety issues (e.g. do not go to other patient rooms). Improved ward atmosphere, simplifying the work in the ward.<br>-No conflicting rules between wards in the same hospital after updating rules in hospital-level | |
| **Lack of alternatives for coercive measures**<br>- Too noisy and crowded environment.<br>-Patients do not have a space where to calm down, relax and have privacy. This may increase in restrictions.<br>- There was no tools in the comfort room for patients to use, to calm down | 6 | -A room that is safe and where you can go to calm down will be created in the ward<br>-Seclusion room will be used for calming down<br>- Comfort rooms will be equipped with e.g. a weighted blanket, stress ball, rubics cube and other tools to reduce anxiety | - Permission is not given to acquire the furniture for comfort room or there are no suitable furniture existing in the storage. | - Comfort room will be used for recreational purposes by patients e.g. to lie and listed to music loud or for self-harm. Patients might therefore start to behave poorly to get to the comfort room | - In some wards, frequency of using the room has been low.<br>- When comfort room is used, reduces conflicts, self-harm, use of medication and frequency and length of seclusion and physical restrictions, and helps reducing anxiety, agitation and insecurity of patients' | - The room has been frequently used as a patient room due to having extra patients. |

© 2022 Välimäki M et al. *JAMA Network Open.*

# eTable 7. Sensitivity analysis

| | Intervention | Control | | | | | Intervention | Control | | | | | | |
|---|---|---|---|---|---|---|---|---|---|---|---|---|---|---|
| | | | | | | | | | | | | | | Adjusted[a] |
| | Baseline, year 2015 | | RR (95% CI) | p-value | Adjusted[a] RR (95% CI) | Adjusted[a] p-value | Year 2017 | | RR (95% CI) | p-value | Adjusted[a] RR (95% CI) | Adjusted[a] p-value | Interaction group×year | Interaction group×year |
| PRIMARY OUTCOMES | | | | | | | | | | | | | | |
| **ORGANISATIONAL OUTCOMES** | | | | | | | | | | | | | | |
| **Seclusion room** | | | | | | | | | | | | | | |
| **All wards included in the anallysis** | | | | | | | | | | | | | | |
| Occurrence of seclusion at ward level/Number of all patients[b] | 629/4163 | 580/4186 | 0.93 (0.41; 2.11) | 0.86 | 0.86 (0.40; 1.82) | 0.68 | 585/4089 | 764/4092 | 0.72 (0.32; 1.63) | 0.42 | 0.66 (0.31; 1.41) | 0.27 | 0.003 | 0.003 |
| (%) | (15.11) | (13.86) | | | | | (14.31) | (18.67) | | | | | | |
| | | | | | | | | | | | | | | |
| Two wards exluded[c] | | | | | | | | | | | | | | |
| Occurrence of seclusion at ward level/Number of all patients[b] | 574/3336 | 580/4186 | 1.16 (0.57; 2.36) | 0.67 | 1.07 (0.58; 1.99) | 0.82 | 559/3294 | 764/4092 | 0.92 (0.45; 1.86) | 0.81 | 0.85 (0.46; 1.58) | 0.60 | 0.007 | 0.009 |
| | (17.21) | (13.86) | | | | | (16.97) | (18.67) | | | | | | |
| | | | | | | | | | | | | | | |
| Two wards excluded[d] | | | | | | | | | | | | | | |
| Occurrence of seclusion at ward level/Number of all patients[b] | 570/3444 | 580/4186 | 0.98 (0.49; 1.93) | 0.94 | 1.03 (0.53; 1.99) | 0.93 | 553/3342 | 764/4092 | 0.79 (0.40; 1.56) | 0.49 | 0.83 (0.43; 1.61) | 0.58 | 0.015 | 0.016 |
| | (16.55) | (13.86) | | | | | (16.55) | (18.67) | | | | | | |
| | | | | | | | | | | | | | | |
| Five wards were exclused[e] | | | | | | | | | | | | | | |
| Occurrence of seclusion at ward level/Number of all patients[b] | 449/2678 | 580/4186 | 1.07 (0.50; 2.27) | 0.86 | 1.14 (0.55; 2.36) | 0.72 | 403/2603 | 764/4092 | 0.78 (0.37; 1.66) | 0.50 | 0.83 (0.40; 1.73) | 0.61 | 0.001 | 0.002 |
| | (16.77) | (13.86) | | | | | (15.48) | (18.67) | | | | | | |
| | | | | | | | | | | | | | | |
| Six wards excluded[f] | | | | | | | | | | | | | | |
| Occurrence of seclusion at ward level/Number of all patients[b] | 337/1822 | 580/4186 | 1.09 (0.49; 2.42) | 0.83 | 1.26 (0.62; 2.58) | 0.51 | 280/1948 | 764/4092 | 0.76 (0.34; 1.69) | 0.48 | 0.90 (0.44; 1.85) | 0.77 | 0.001 | 0.002 |

© 2022 Välimäki M et al. *JAMA Network Open.*

**eAppendix 3. Evidence existing prior to this study**

Staff education for the reduction of coercive measures is widely supported by professional associations, but fewer evidence-based and tested educational protocols supporting optimising outcomes are available. On-the-job training is widely used in psychiatric hospitals although its effectiveness has seldom been evaluated with randomised controlled trials. We designed the study in 2017, when no systematic reviews had been published, and only a few evaluations of the effectiveness of non-pharmacological studies to reduce the use of coercive measures had been published, to our knowledge. In our large-scale study, we aimed to design an educational intervention using an evidence-based pathway for how to make changes using systematic identification of the views of patients, family members and staff regarding current problems and shared solutions to the problems using evidence-based approaches to reduce the use of coercive measures on psychiatric wards.

We searched APA PsycINFO (Ebscho) and PubMed (Medline) for randomised controlled trials published between 1 January 1981 and 27 May 2022 that reported on psychiatric hospital non-pharmacological interventions targeting the reduction of the use of any type of coercive methods for people with any type and severity of mental illness using the terms: (hospital* OR psychiatric* OR 'mental health') AND ('coercive measure*' OR 'coercive method*' OR coercive OR coercion OR seclusion OR restraint OR 'forced injection' OR 'forced medication') AND ('randomised control* trial*' OR RCT OR 'randomised controlled trial'), with no language restrictions. Our search identified 341 citations, of which five were cluster trials conducted in a psychiatric hospital setting.

All interventions included multiple components focusing on leadership, modification of the environment, repeated violence risk assessment, or patient and family involvement. Three cluster randomised studies identified interventions that decreased patients' seclusion-restraint time[1-3] while in two other studies, the intervention decreased the use of seclusion-restraint or containment methods.[4,5].

© 2022 Välimäki M et al. *JAMA Network Open.*

One study[3] included self-reflective exercises in their intervention to help staff members to manage patients' challenging behaviour on the wards and showed that the three-month intervention reduced the lengths of patient mechanical restraints during the seclusion period. Two studies[1,4] used structured risk assessment methods to assess patient violence on daily basis. As an outcome, one study[4] showed a significant decrease in coercive measures used, and another study[1] showed a significant decrease in the lengths of patient seclusions. One study[2] used multiple methods to combine post-incident analysis and a review of the problems and coercion incidences, staff counselling and discussions, and tailored crisis plans for patients. This one-year intervention was found to be effective in reducing days where seclusion, restraint and room observations occurred, and decreasing the lengths of seclusion and restraint. In addition, one intervention[5] included a package of ten interventions used in a three-month period (e.g., standards of behaviour, a de-escalation model for staff, sharing good things about each patient, inter-patient support meetings, distraction and sensory modulation tools to use with agitated patients). The intervention was effective in reducing the use of coercive measures.

However, all five studies employed a cluster randomized design, but failed to include cluster effects in their estimates.

**Added value of this study**

Our study tested an educational intervention where an evidence-based pathway was used to decrease the use of seclusion rooms in psychiatric hospitals. This process was led by the research personnel, but changes implemented on study wards were implemented by nurses. The educational intervention combined evidence-based knowledge shared with nurses, user-centred approaches focusing on the needs of patients, family members, and nurses based on lived experiences in clinical practice. Responses to these problems were solved using evidence-based, tailored treatment methods. The educational intervention with multiple components had only a weak effect in reducing the use of seclusion rooms on psychiatric wards. At the same time, the use of forced medication increased partially. Despite the strong engagement of various stakeholders in the study, no changes were found in nurses' team climate or patient-related outcomes.

© 2022 Välimäki M et al. *JAMA Network Open.*

**Implications of all the available evidence**

Considering all the available evidence, multicomponent educational interventions for nurses could reduce the use of seclusion rooms and the number of patient restrictions in psychiatric hospitals, but other form of coercion, such as the use of forced medication may concurrently increase. These multicomponent educational packages with more focused intervention elements toward coercion practices could have some potential to shift practice and treatment culture toward less coercive care at least in Europe. Whether this is evident in middle- and low- income countries should be tested more widely. Therefore, the results of this trial have opened the doors to very large trials across the world. In addition, as the available evidence today focuses on short-term outcomes, future research is needed to test the best combinations of intervention components to reduce all types of coercion with lasting effects.

**eReferences**


1 van de Sande R, Nijman H, Noorthoorn E, *et al*. Aggression and seclusion on acute psychiatric wards: effect of short-term risk assessment. *BMJ* 2011; **199**: 473–78.

2 Putkonen A, Kuivalainen S, Louheranta O, *et al*. Cluster-randomizedised controlled trial of reducing seclusion and restraint in secured care of men with schizophrenia. *Psychiatr Serv* 2013; **64**: 850–55.

3 Kontio R, Pitkänen A, Joffe G, Katajisto J, Välimäki M. eLearning course may shorten the duration of mechanical restraint among psychiatric inpatients: a cluster-randomized trial. *Nord J Psychiatry* 2014; **68**: 443–49.

4 Abderhalden C, Needham I, Dassen T, Halfens R, Haug HJ, Fischer JE. Structured risk assessment and violence in acute psychiatric wards: randomised controlled trial. *Br J Psychiatry* 2008; **193**: 44–50.

5 Bowers L, James K, Quirk A, Simpson A; SUGAR, Stewart D, Hodsoll J. Reducing conflict and containment rates on acute psychiatric wards: The Safewards cluster randomised controlled trial. *Int J Nurs Stud* 2015; **52**: 1412–22.


© 2022 Välimäki M et al. *JAMA Network Open.*
