# Supplementary material for: An Evidence-Based Educational Intervention for Reducing Coercive Measures in Psychiatric Hospitals: A Randomized Clinical Trial
Source: JAMA Netw Open. 2022 Aug 30;5(8):e2229076. doi: 10.1001/jamanetworkopen.2022.29076 (PMC9428738; doi:10.1001/jamanetworkopen.2022.29076)
Supplement: Supplement 3. — Data Sharing Statement [file jamanetwopen-e2229076-s003.pdf]

Data Sharing Statement

Välimäki. An Evidence-Based Educational Intervention for Reducing Coercive Measures in Psychiatric Hospitals. *JAMA Netw Open*. Published August 30, 2022. doi:10.1001/jamanetworkopen.2022.29076

Data
Data available: No

Additional Information
Explanation for why data not available: Individual participant data will not be available due to ethical reasons.
